# Hypoxia and its implications in rheumatoid arthritis

**DOI:** 10.1186/s12929-016-0281-0

**Published:** 2016-08-22

**Authors:** Celia María Quiñonez-Flores, Susana Aideé González-Chávez, César Pacheco-Tena

**Affiliations:** 1 Facultad de Medicina y Ciencias Biomédicas, Universidad Autónoma de Chihuahua, Circuito No.1, Nuevo Campus Universitario, Chihuahua, C.P. 31240 México; 2 Facultad de Ciencias de la Cultura Física, Universidad Autónoma de Chihuahua, Circuito No.1, Nuevo Campus Universitario, Chihuahua, C.P. 31240 México

**Keywords:** Hypoxia-inducible factor (HIF), Autoimmune disease, Inflammation

## Abstract

Alterations in tissue oxygen pressure contribute to a number of diseases, including rheumatoid arthritis (RA). Low partial pressure of oxygen, a condition known as hypoxia, is a relevant feature in RA since it is involved in angiogenesis, inflammation, apoptosis, cartilage degradation, energy metabolism, and oxidative damage. Therefore, alterations in hypoxia-related signaling pathways are considered potential mechanisms of disease pathogenesis. The objective of this review is to highlight and update our current knowledge of the role of hypoxia in the pathogenesis of RA. We describe the experimental evidence that RA synovial tissue exists in a hypoxic state, as well as the origin and involvement of synovial hypoxia in different aspects of the pathogenic process.

## Background

Rheumatoid arthritis (RA) is a severe chronic autoimmune disease characterized by joint inflammation and destruction and the presence of autoantibodies (rheumatoid factor and anti-citrullinated protein antibodies). RA affects 1 % of the world’s population [1], and its prevalence among Mexicans is 1.6 % [2]. RA inflames the synovial membrane of diarthrodial joints and damages the articular tissues, leading to severe functional disarrangement of the entire joint. The initial stages of RA synovitis are characterized by proliferation of the microvasculature and secondary edema. Eventually, this process matures into a progressive infiltration of immune cells, including B cells, T cells, and monocytes, from the bloodstream. These immune cells are activated in the joint, differentiate, and acquire mature phenotypes. The influx of immune cells is also associated with phenotypic changes in synoviocytes, the typical resident cells. Both fibroblast- and monocyte-derived synoviocytes proliferate extensively and participate in the inflammatory process. In the latter stages of disease, the synovium becomes a mass of growing tissue, called *pannus*, which has the capacity to overlay, degrade, and invade the cartilage and resorb the bone [3]. In addition, RA patients develop systemic complications such as vasculitis and cardiovascular, pulmonary, skeletal, and psychological disorders. The etiology of RA remains elusive; however, there is evidence that both genetic and environmental factors are involved [4].

Unexpectedly, synovial hypoxia (defined as low oxygen partial pressure, pO_2_) is a constant feature of RA. Hypoxia can induce angiogenesis, inflammation, apoptosis, cartilage erosion, abnormal energy metabolism, and oxidative damage. Synovial hypoxia is thus considered a potential pathogenic factor in RA.

The aim of this review is to highlight and update our understanding of the potential roles of hypoxia in the pathogenesis of RA. We describe the evidence supporting the existence of hypoxia in the synovial tissue, its origin, and the potential connections between synovial hypoxia and the cells, soluble mediators, and processes currently considered relevant to the pathogenesis of RA.

## Review

### Hypoxia and signaling mediators

Atmospheric oxygen is extracted and transported to tissues via the blood. Under normal circumstances, the pO_2_ is not uniform across tissues but varies in the different body compartments. The pO_2_ can reach 100 mm Hg (16 % O_2_) in the pulmonary alveoli but decreases to ~40 mm Hg (6 % O_2_) in most tissues [5].

Tissue hypoxia occurs when there is an imbalance between the supply and demand for oxygen and can result in cell dysfunction and even death. Under hypoxic conditions, cells activate mechanisms to respond and adapt to this environment. One of the key regulators of the response is the transcription factor hypoxia-inducible factor (HIF) [6], which regulates genes related to angiogenesis, apoptosis, cell migration, vasomotor control, erythropoiesis, pH regulation, energy metabolism, and many other processes [7].

**Fig. 1:**
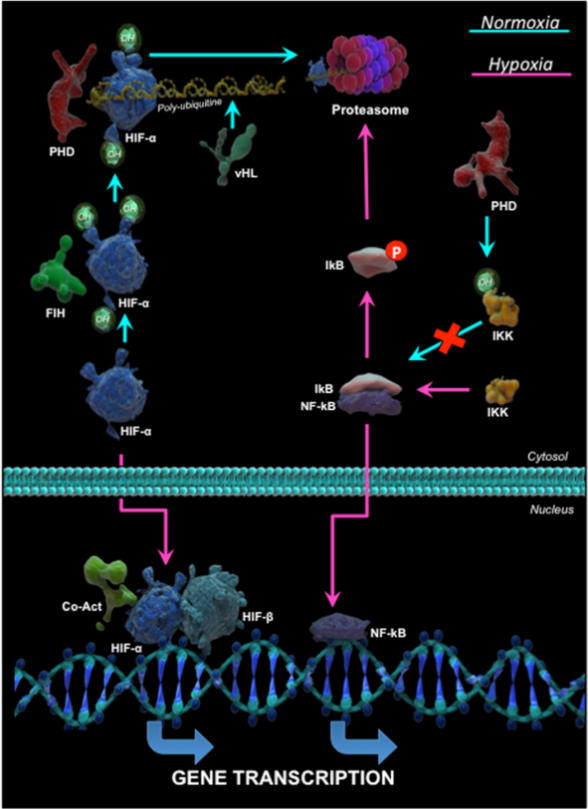
*Hypoxia and signaling mediators*. Representation of the signaling pathways mediated by NF-kB and HIF under normoxic and hypoxic conditions. Under normoxic conditions, HIF-α is hydroxylated by PHD and FIH enzymes to promote its ubiquitination and proteasomal degradation. During hypoxia, stabilized HIF-α relocates to the nucleus where it forms a dimer with HIF-β, recruits coactivators, and initiates transcription of hypoxia-regulated genes. NF-kB is inactive in the cytoplasm owing to its association with IkB. Activation of this pathway is regulated by IKK, which mediates the phosphorylation and degradation of IkB, allowing NF-kB to translocate to the nucleus where it activates gene transcription. PHD has been proposed to inhibit IKK under normoxic conditions. CoAct, coactivators; FIH, factor-inhibiting HIF (asparagyl β-hydroxylase); HIF, hypoxia-inducible factor; IkB, inhibitory protein; IKK, IkB kinase; NFkB, nuclear factor-kappa B; PHD, prolyl hydroxylase; vHL, von Hippel Lindau tumor suppressor

HIF was first detected in a nuclear extract from the human hepatoma cell line Hep3B [6] and was later described as a heterodimeric transcription factor composed of a HIF-1α subunit, which is regulated by oxygen levels, and a HIF-1β subunit, which is expressed constitutively [8]. Both subunits are members of a subfamily of factors with a basic helix-loop-helix (bHLH) domain and a Per, ARNT, and Sim (PAS) domain [9]. To date, three isoforms of the HIF-α subunit have been described—HIF-1α, HIF-2α, and HIF-3α—of which HIF-1α is the most studied. The three isoforms are characterized by the presence of bHLH, PAS, and oxygen-dependent degradation (ODD) domains. HIF-1α and HIF-2α share a number of structural and functional similarities, but HIF-1α is ubiquitously expressed, whereas HIF-2α is restricted to certain cell types and, in some cases, mediates different biological functions [10]. HIF-α protein levels are regulated at the stability and synthesis levels. Under normoxic conditions, HIF-α is modified by prolyl hydroxylases (PHD), which require ferrous iron (Fe^2+^) as an enzymatic cofactor and α-ketoglutarate and oxygen as substrates. The three forms of PHD (PHD-1, -2, and -3) differ in their affinities for the 1α, 2α, and 3α isoforms of HIF [11, 12]. PHD hydroxylates proline residues in the ODD domain of HIF-α [13]. The hydroxylated residues are subsequently recognized by the tumor suppressor von Hippel Lindau (vHL) protein, which recruits the Elongin-C–Elongin-B–Cullin 2–E3 ubiquitin ligase complex, leading to polyubiquitination and proteasomal degradation of HIF-α [14]. The activity of HIF-α is also regulated through hydroxylation of asparagine residues in its C-terminal transactivation domain by asparagyl β-hydroxylases (factor-inhibiting HIF), which are also dependent on oxygen, α-ketoglutarate and Fe^2+^. In this case, hydroxylation prevents HIF-α from interacting with its coactivator [15–17]. During hypoxia, these oxygen-dependent hydroxylation reactions are not performed, and HIF-α does not bind to vHL. HIF-α is thus stabilized and concentrates in the cytoplasm, subsequently translocating to the nucleus where it dimerizes with HIF-β. The HIF-αβ heterodimer binds to specific DNA sequences known as hypoxia response elements, and finally, recruitment of coactivators enables transcription of HIF-dependent genes (Fig. 1). Recent work has shown that PHD-2 is the major hydroxylase regulating HIF-α levels and the expression of angiogenic genes in fibroblasts derived from patients with RA [18].

Although a role for HIF in RA pathogenesis has been inferred, the precise mechanisms by which this occurs remain to be defined. In the past, little was known about the contributions of the different HIF-α isoforms, but recent studies have shown that HIF-2α plays a fundamental role in RA independently of HIF-1α. HIF-2α is mainly overexpressed in fibroblasts, where it enhances their osteoclastogenic potential and regulates cell proliferation, expression of receptor activator of nuclear factor kB ligand, and induction of several catabolic factors [19]. The HIF-2α isoform is also involved in cartilage destruction. In a mouse arthritis model, HIF-2α–induced chemokine production by chondrocytes stimulated the migration and invasion of synovial fibroblasts, leading to cartilage erosion [20].

Hypoxia is not the only condition to activate and stabilize HIF-α. Physical factors such as heat and low pH, and biochemical factors such as hormones, cytokines, growth factors, and reactive oxygen species (ROS), may also induce and activate HIF-α. Bacterial lipopolysaccharides have been reported to induce HIF-1α activation in human monocytes and macrophages via nuclear factor-kappa B (NF-kB) and p44/42 mitogen-activated protein kinase (MAPK) pathways [21]. Under normoxic conditions, proinflammatory cytokines such as tumor necrosis factor (TNF)-α and interleukin (IL)-1β can increase HIF expression at the mRNA and protein levels in synovial and gingival fibroblasts through the MAPK and phosphoinositide 3-kinase pathways [22, 23].

In addition to its effects on HIF-1α, hypoxia can activate signaling through members of the NF-kB family, which are considered the main proinflammatory transcription factors. NF-kB is overexpressed in RA synovial tissue [24] and regulates the expression of genes such as TNF-α, IL-6, IL-8, various chemokines, vascular endothelial growth factor (VEGF), and matrix metalloproteinase (MMP) 1, 3, and 13, which are associated with inflammation, angiogenesis, and tissue destruction [25]. NF-kB therefore acts in an additive and synergistic manner with HIF to contribute to the maintenance of the inflammatory response [26, 27]. In the classical pathway of NF-kB activation, NF-kB is retained in the cytoplasm by binding to the inhibitory protein IkB. Cell stimulation activates IkB kinase (IKK), which phosphorylates IkB and promotes its degradation. The release from IkB thus allows NF-kB to translocate to the nucleus where it binds to specific DNA sequences (kB sites) to activate transcription of genes appropriate to the activating stimulus. It has been proposed that PDHs hydroxylate and inhibit IKK activity under normoxic conditions [27], but under hypoxic conditions, the lack of hydroxylation permits IKK to phosphorylate and promote the degradation of IkB, leading to activation of NF-kB (Fig. 1).

### Hypoxia in RA

#### Evidence of hypoxia in RA synovial tissue

Hypoxia is a feature of the synovial membrane in RA. This was first detected in 1970, when the oxygen tension in the synovial fluid of RA patients was found to be lower than that in healthy controls and osteoarthritis patients [28, 29]. Subsequently, tissue oximeters were used to confirm that hypoxia is a characteristic of RA synovial tissue [30, 31] and correlates with the intensity of the inflammatory process and cell migration [31].

Other studies have reported an inverse relationship between oxygen tension and synovial fluid volume [32] associated with acidosis, low glucose concentrations, high lactic acid concentrations [33, 34], and increased activity of some glycolytic enzymes, indicating that anaerobic metabolism is promoted as a survival mechanism within the hypoxic environment of the synovium [34].

#### Origins of hypoxia in RA

Two main theories have been proposed to explain hypoxia in RA synovial tissue. The first suggests that increased cell proliferation generates a high metabolic demand and increases the distance between proliferating cells and nearby blood vessels, which cannot meet the tissue oxygen requirements [35]. The second posits that the increased intraarticular pressure resulting from hyperplasia, synovial fluid effusion, and joint movements within the rigid joint capsule intermittently collapse the network of capillaries and therefore decrease the blood flow in the synovial tissue [36].

Another potential explanation for synovial hypoxia could be the enhanced expression of angiotensin converting enzyme (ACE), a membrane metalloprotease that catalyzes the formation of angiotensin II from its inactive precursor, angiotensin I. Angiotensin II is a potent vasoconstrictor and ACE is overexpressed in stromal cells in RA synovial tissue, generating local vasoconstriction and enhancing hypoxia [37].

Hypoxia might play a role not only in perpetuating synovial inflammation in RA but also in inducing it, as suggested by work from Jeon and colleagues [38] using a murine model of collagen-induced arthritis (CIA). Hypoxia levels were evaluated from the early stages of the disease and related to clinical aspects of the inflammatory process. Interestingly, in this model, hypoxia was present from the pre-arthritic stages. From the perspective of these findings, in which synovial hypoxia is detected before clinical onset of arthritis, the two theories mentioned earlier become insufficient to explain the origin of hypoxia in RA. It is likely that cell proliferation and intraarticular high pressure explain only the maintenance of the hypoxic state once the inflammatory process is established in the tissue. This highlights the importance of exploring the pre-arthritic phases in relation to the onset of the hypoxic state, for which signaling mediators have already been demonstrated in the arthritic phase.

#### Hypoxia, inflammation, and angiogenesis in RA

The first evidence that HIF-1α participates in the inflammatory process came from Cramer et al. [39], who showed that deletion of HIF-1α in macrophages reduces disease severity in different models of acute and chronic inflammation (including a passively induced arthritis model). Moreover, the bactericidal capacity of these macrophages was also decreased by HIF-1α deletion [39, 40].

HIF-1α is upregulated in RA fibroblasts [41], CD3^+^ T cells [42], and CD68^+^ synovial macrophages [43]. The latter cells have been shown to express several hypoxia-regulated genes in RA, including stromal cell-derived factor-1 (SDF-1), IL-8, VEGF, IL-1β, and TNF-α [44]. In addition, HIF-2α nuclear and cytoplasmic expression has also been demonstrated in the synovial lining and stromal cells of patients with RA and osteoarthritis [45].

SDF-1 is a potent angiogenic and chemotactic factor that is upregulated in synovial fibroblasts and promotes the recruitment of CXCR4+ ly mphocytes to the joints in RA. Expression of SDF-1 and VEGF in RA synovial tissue is hypoxia dependent [41], and CXCR4 expression can be induced by hypoxia. Celastrol, a triterpine compound with antioxidant and anti-inflammatory activity, inhibits hypoxia-induced migration and invasion of synovial fibroblasts via suppression of HIF-1α-mediated CXCR4 expression [46]. The increased survival of T cells under hypoxic conditions is another factor that contributes to their accumulation in the synovium [42, 47]. Additionally, HIF-1α overexpression is involved in the regulation of apoptosis in neutrophils [48, 49].

Hypoxia and IL-17 have a synergistic effect on the migration and invasion of synovial fibroblasts by increasing the expression of MMP2 and MMP9 via the NF-kB–HIF-1α pathway [50]. HIF-1α also promotes the activation of signaling pathways and controls IL-33 production by fibroblasts, which in turn induces expression of HIF-1α and generates a regulatory cycle that perpetuates inflammation in RA [51]. A recent study found evidence for a functional interaction during HIF-1α, Notch-3, and STAT-1 to regulate proinflammatory mechanisms in RA synovial fibroblasts during hypoxia [52].

Hypoxia is also associated with the differentiation of some synovial cells. Differentiation of Th0 lymphocytes towards Th17 cells, which are important for the development of autoimmune diseases such as RA, is associated with HIF-1α–regulated expression of the glucose transporter GLUT-1 [53]. In contrast, hypoxia inhibits the expression of several differentiation and maturation markers in dendritic cells, and reduces their ability to stimulate T cells and upregulate the production of proinflammatory cytokines such as TNF and IL-1β [54].

Toll-like receptors (TLRs) sense highly conserved structural motifs found in many molecules of bacterial and viral origin. Recognition of these molecules (e.g., bacterial lipoproteins, double-stranded RNA, and lipopolysaccharides) by TLRs upregulates costimulatory molecules and induces the production of proinflammatory and tissue-destructive mediators. Many cells within the joint express TLRs, and they can be activated by a variety of endogenous TLR ligands present within the inflamed RA joints. Current evidence suggests that TLR activation may contribute to the persistent expression of proinflammatory cytokines in RA through activation of NF-kB [55]. Stimulation of synovial fibroblast TLRs leads to increased expression of inflammatory cytokines, MMPs, and adhesion molecules. Moreover, induction of HIF-1α by hypoxia synergizes with TLR signaling to produce inflammatory cytokines (IL-6, IL-8, TNF-α), MMP-1, -3, -9, and VEGF, thereby exacerbating RA [56]. NFkB links TLR signaling to the hypoxic response through transcriptional regulation of HIF-1α, providing a likely explanation for these observations.

High-mobility group box 1 (HMGB-1), a non-histone nuclear protein, is an endogenous TLR ligand and has been detected in the synovial fibroblasts of RA patients. HMGB-1 stimulates the expression of VEGF, and this is attenuated by inhibition of HIF-1α. Similarly, treatment of CIA mice with a neutralizing anti-HMGB-1 prevents the formation of blood vessels, which is associated with a decrease in HIF-1α expression in the synovial tissue. This suggests that HMBG-1 participates in the process of HIF-1-mediated angiogenesis [57].

Stimulation of angiogenesis in hypoxic conditions is part of the cellular effort to restore oxygen delivery to the tissue. HIF-1α and HIF-2α isoforms are expressed in the RA synovium at levels related to the magnitude of the angiogenic response [58].

VEGF is one of the most potent and well-studied angiogenic stimuli, and VEGF mRNA levels are increased by HIF-1α during hypoxia. VEGF contributes to the initial vasodilation through induction of nitric oxide and increased endothelial cell permeability. In addition, VEGF prevents apoptosis of endothelial cells and stimulates their proliferation and migration [59]. Hypoxia also induces the expression of other angiogenic factors such as IL-8 [60], CCL20 [61], and SDF1 [41, 62].

**Fig. 2:**
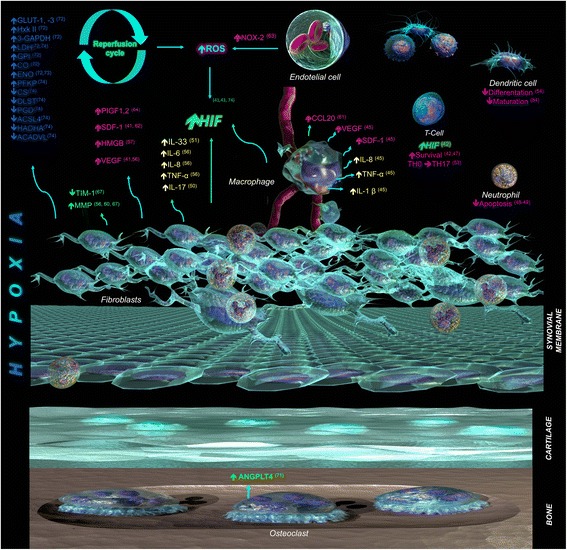
*Direct and indirect effects of hypoxia and*/*or HIF on the different cell types contributing to RA pathogenesis*. References relevant to the indicated phenomena are provided in the scheme. The colors of the molecules name indicate if the molecule has effect on: inflammation (yellow), oxidative stress (cyan), energy metabolism (*blue*), angiogenesis (*magenta*), destruction (*green*). ACADVL very long-chain acyl-CoA dehydrogenase; ACSL, long-chain fatty acid-CoA ligase; ANGPLT, angiopoietin-like; CCL20, chemokine (C-C motif) ligand 20; CO, mitochondrial cytochrome; CS, citrate synthase; DLST, oxoglutarate dehydrogenase complex component E2; ENO, enolase; GAPDH, glyceraldehyde 3-phosphate dehydrogenase; GLUT, glucose transporter; GPI, glucose phosphate isomerase; HADHA, hydroxyacyl-CoA dehydrogenase/3-ketoacyl-CoA thiolase/enoyl-CoA hydratase; HIF, hypoxia-inducible factor; HMGB, high-mobility group protein B; Hxk, hexokinase; IL, interleukin; LDH, lactate dehydrogenase; MMP, matrix metalloproteinase; NOX, NADPH oxidase; PFKP phosphofructokinase; PGD, phosphogluconate dehydrogenase; PLGF, placental growth factor; ROS, reactive oxygen species; SDF, stromal cell-derived factor; TIMP, tissue inhibitor of metalloproteinase; TNF, tumor necrosis factor; VEGF, vascular endothelial growth factor

NADPH oxidase enzyme-2 (NOX2) is overexpressed in the joints of RA patients and those of rats in the CIA model of arthritis. This enzyme generates superoxide by transferring electrons from NADPH inside the cell across the plasma membrane to molecular oxygen to produce superoxide anion, a reactive free radical. NOX-2 is overexpressed in RA joints in inverse proportion to the pO_2_. NOX-2 activators and 3 % hypoxia promote human microvascular endothelial cell migration, tubule formation, and secretion of proangiogenic mediators, suggesting that oxidative stress derived from the hypoxia-induced increase in NOX-2 activity is an initiating factor in angiogenesis [63]. The placental growth factors PlGF-1 and PlGF-2 are also hypoxia-inducible angiogenic growth factors and promote the proliferation, survival, migration, and invasion of RA synovial fibroblasts [64]. In a rat arthritis model, synovial angiogenesis was associated with overexpression of VEGF, CD34, and HIF-1α, and the levels of HIF-1α were positively correlated with the arthritis index [65] (Fig. 2).

Although angiogenesis is increased in RA, the formation of new blood vessels fails to keep pace with the rapid thickening caused by synovial tissue hyperplasia. Moreover, the presence of immature blood vessels in the synovial tissue may explain the increased density of blood vessels and persistent hypoxia in the RA synovium [66].

#### Hypoxia and cartilage destruction in RA

The destruction of articular cartilage in RA is associated with increased activity of MMPs. These enzymes degrade extracellular matrix proteins, and their activity is regulated by endogenous tissue inhibitors of MMPs (TIMPs). MMP expression is elevated during repair and remodeling of damaged tissues. RA synovial fibroblasts exposed to hypoxia express increased levels of MMP-1 and MMP-3 and decreased levels of TIMP-1 at both the mRNA and protein levels [60, 67].

The MMPs promote remodeling of the extracellular matrix during angiogenesis, and since their expression is mainly induced by Ets-1, this transcription factor is considered to be involved in the invasion and destruction of cartilage and bone in RA [68]. Ets-1 is inducible by hypoxia [69] and co-localizes with HIF-1α in the synovial inflammatory infiltrate in an adjuvant-induced arthritis model in rats [70].

Angiopoietin-like 4 (ANGPTL4) stimulates bone resorption mediated by osteoclasts, which are the cells responsible for bone loss in RA. ANGPLT4 is overexpressed in RA osteoclasts in a HIF-1α–dependent manner. Other cell types in RA synovial tissue also overexpress ANGPLT4, suggesting that there are several sources of ANGPLT4 to promote bone resorption [71] (Fig. 2).

#### Hypoxia and energy metabolism in RA

Energy metabolism has recently become an important area of study in RA, and hypoxia is known to induce substantial metabolic changes in RA synovial tissue. Under aerobic conditions, the oxidation of one molecule of glucose to carbon dioxide (CO_2_) produces ~36 molecules of adenosine triphosphate (ATP), and this is the most efficient route of energy generation. However, anaerobic glycolysis is much less efficient and produces only two molecules of ATP during conversion of one molecule of glucose to lactate. Anaerobic glycolysis is relevant to conditions associated with a restricted oxygen supply, such as the hypoxia observed in RA joints. Indeed, the increased activity of some glycolytic enzymes, diminished glucose concentrations, and elevated lactate concentrations in RA synovial tissue indicate that anaerobic glycolysis is favored in this hypoxic environment.

Under hypoxic conditions, HIF-1α increases the expression of the glucose transporters GLUT1 and GLUT3 to enhance glucose uptake, and it regulates the expression of hexokinase II, glyceraldehyde 3-phosphate dehydrogenase, lactate dehydrogenase (LDH), and mitochondrial cytochrome oxidase to enhance glycolysis in the RA synovial tissue. HIF-1α also upregulates glucose phosphate isomerase, which together with enolase, aldolase, and triose phosphate isomerase, act as autoantigens [72]. Therefore, during its attempt to increase energy production for cell survival, HIF also generates antigenic targets and promotes autoimmunity. Moreover, upregulation of α-enolase in RA fibroblasts during hypoxia is associated with hyperproliferation and overexpression of anti-apoptotic proteins, such as Bcl-2, survivin, and cyclin B1 [73].

Interestingly, a recent study confirmed that concentrations of glucose and lactate are decreased and increased, respectively, in the synovial fluid of RA patients [74]. Levels of glucose-1-phosphate and D-mannose (glycolytic donors) were also decreased. In these patients, enzymes involved in anaerobic catabolism were upregulated (6-phosphofructokinase type C [PFKP] and LDH A), and enzymes involved in aerobic oxidation and fatty acid oxidation were downregulated (citrate synthase [CS], 2-oxoglutarate dehydrogenase complex component E2 [DLST], 6-phosphogluconate dehydrogenase [PGD], long-chain fatty acid-CoA ligase 4 [ACSL4], very long-chain acyl-CoA dehydrogenase [ACADVL], and hydroxyacyl-CoA dehydrogenase/3-ketoacyl-CoA thiolase/enoyl-CoA hydratase [HADHA]). CS and DLST are the rate-limiting enzymes of the tricarboxylic acid cycle; therefore, the significantly reduced expression of these enzymes together with the decreased levels of citric acid suggest that ATP production through aerobic oxidation is decreased in RA synovial fluid. Furthermore, expression of HIF-1α, PFKP, and LDHA is decreased and expression of CS, DLST, PGD, ACSL4, HADHA, and ACADVL is increased in synovial fibroblasts after HIF-1α knockdown. Collectively, these results suggest that HIF plays a significant role in regulating the metabolic flux (Fig. 2).

Protein citrullination may also be associated with hypoxia. α-Enolase and peptidylarginine deiminase type II, which are upregulated in RA fibroblasts under hypoxic conditions, have known links to citrullinated antigens. This link between hypoxia and citrullination has been described in astrocytes, suggesting that it might also exist in the RA synovium [75].

#### Hypoxia and reactive oxygen species in RA

Synovial perfusion is directly affected by the high intraarticular pressure prevailing in the synovial tissue of RA. This pressure may be further increased by movement, which contributes to local tissue hypoxia followed by reoxygenation when the joint is unloaded. These repeated cycles of hypoxia–reoxygenation lead to increased levels ROS and consequently to NF-kB activation [76]. Experimental evidence suggests that ROS produced by the NOX system maintains HIF-1α in an inactive state. Under hypoxic conditions, insufficient amounts of ROS are formed and HIF-1α is activated [77]. In contrast, ROS produced by mitochondria during hypoxia have a stabilizing effect on HIF-1α [78, 79]. Although ROS have several physiological functions in the cell, an imbalance between oxidant and antioxidant activity can lead to a state of oxidative stress. Such stress affects many cellular components, including DNA, lipids, and proteins, causing changes in their structures and functions, as is observed in RA (Fig. 2).

## Conclusions

The pathogenesis of RA is incompletely understood. Although inflammatory and autoimmune phenomena are believed to be the central abnormalities, the precise etiology remains elusive. Synovial hypoxia is a consistent finding and can be linked to several pathogenic processes through direct and indirect effects on angiogenesis, cartilage damage, bone resorption, oxidative damage, and inflammation. It should be noted, however, that hypoxia is not the only promoter of inflammation in RA, and it can be induced and perpetuated by diverse stimuli. Nevertheless, the induction of an anaerobic glycolytic phenotype in the synovium is specifically induced by hypoxia. Once anaerobic glycolysis is established in the synovium, several of the enzymes induced by this metabolic shift may become antigenic. Hypoxia modifies the metabolic environment in the synovium, but in the context of cellular stress, presentation of the upregulated antigenic enzymes could initiate an autoimmune response. For example, glucose phosphate isomerase, a glycolytic enzyme induced by hypoxia, is autoantigenic. The possible involvement of hypoxia in the citrullination process has also been proposed.

Overall, hypoxia is an interesting condition with effects that reflect a complex mechanism of action in RA. This requires further study, especially in the pre-arthritic stages when hypoxia is already present, according to the results of Jeon and colleagues. These findings provide new perspectives to understand the pathogenesis of RA and to identify new therapeutic targets.

## Abbreviations

ACADVL: Very long acyl-CoA dehydrogenase
ACE: Angiotensin converting enzyme
ACSL: Long chain fatty acid-CoA ligase
ANGPTL: Angiopoietin-like
ATP: Adenosine triphosphate
bHLH: Basic helix-loop-helix
CIA: Collagen-induced arthritis
CO: Mitochondrial cytochrome
CO2: Carbon dioxide
CS: Citrate synthase
DLST: Oxoglutarate dehydrogenase complex component E2
ENO: Enolase
Fe2+: Ferrous iron
FIH: Factor-inhibiting HIF
GAPDH: Glyceraldehyde 3-phosphate dehydrogenase
GLUT: Glucose transporter
GPI: Glucose phosphate isomerase
HADHA: Hydroxyacyl-CoA dehydrogenase/3-ketoacyl-CoA thiolase/enoyl-CoA hydratase
HIF: Hypoxia inducible factor
HMGB: High-mobility group box
Hxk: Hexokinase
IKK: IkB kinase
IL: Interleukin
LDH: Lactate dehydrogenase
MAPK: Mitogen-activated protein kinase
MMP: Metalloproteinase
NADPH: Nicotinamide adenine dinucleotide phosphate
NF-kB: Nuclear factor-kappa B
NOX: NADPH oxidase enzyme
ODD: Oxygen-dependent degradation
PAS: Per, ARNT and Sim
PFKP: Phosphofructokinase
PGD: Phosphugluconate dehydrogenase
PHD: Prolyl hydroxylases
PIGF: Placental growth factor
pO2: Oxygen partial pressure
RA: Rheumatoid Arthritis
ROS: Reactive oxygen species
SDF: Stromal cell-derived factor
TIM: Tissue inhibitors of metalloproteinases
TLR: Toll-like receptors
TNF: Tumor necrosis factor
VEGF: Vascular endotelial growth factor
vHL: von Hippel Lindau
